# Papillary thyroid microcarcinoma: the significance of high risk features

**DOI:** 10.1186/s12885-017-3120-0

**Published:** 2017-02-16

**Authors:** Nori L. Bradley, Sam M. Wiseman

**Affiliations:** Department of Surgery, St. Paul’s Hospital & University of British Columbia, St. Paul’s Hospital Department of Surgery, Room C303, Burrard Building, 1081 Burrard Street, Vancouver, V6Z 1Y6 BC Canada

**Keywords:** Thyroid Cancer, Thyroid Carcinoma, Papillary Carcinoma, Microcarcinoma, Prognosis

## Abstract

**Background:**

Papillary carcinomas that measure 1.0cm or less are diagnosed as papillary thyroid microcarcinomas (PTMs). The clinical significance and recommendations for management of these PTMs is still evolving. The objective of the study was to compare the characteristics of small (<5mm) to large (≥ 5mm) papillary thyroid microcarcinomas.

**Methods:**

Amongst 1459 sequential patients undergoing thyroid surgery at a single center, 132 (9%) cases were diagnosed with PTM. We performed a retrospective analysis of these cases using Fisher’s Exact Test. The statistical significance was set at *p* < 0.05 a priori.

**Results:**

A relationship between large PTM and high risk features was observed only for extra-thyroidal cancer extension (ETE). Six of 57 large PTM (11%) but none of the 75 small PTM had ETE (*p* < 0.01). Lymph node metastases were associated with both small PTM (5/9 cases) and large PTM (4/9 cases). A distant metastases was diagnosed in association with a small PTM.

**Conclusions:**

For PTM, neither small cancer size, nor the absence of high-risk features, excluded the possibility of synchronous lymph node metastases.

## Background

Multiple reports have identified a rising incidence of thyroid cancer over the past several decades, primarily due to the diagnosis of small papillary carcinomas [1, 2]. According to the World Health Organization, papillary carcinomas that measure 1.0cm or less are diagnosed as papillary thyroid microcarcinomas (PTMs) [3]. Recent work has suggested that PTMs are the most commonly diagnosed thyroid cancers in individuals who are older than 45 years, and accounts for almost one quarter of new diagnoses [1]. The clinical significance and recommendations for management of these PTMs is still evolving. In general, PTM is considered a clinically very low risk cancer type that uncommonly causes mortality, with a greater than 90% disease-free survival after long term follow up [2, 4, 5]. Reported rates for distant metastases and mortality from PTM are <0.05% [6]. However, some PTMs exhibit aggressive behaviour, including the development of a regional and distant metastases, and even death [7, 8].

The rising incidence of PTM, and its associated morbidity, requires risk stratification and guidelines for treatment. Currently, treatment of PTM is based upon stratification of cancer risk for aggressive behaviour, which is based on patient and cancer characteristics [2, 4]. Cancers considered high risk include those that are: multifocal, have extrathyroidal extension, have vascular invasion, are incompletely resected, have lymph node metastases, especially if multiple nodes are involved, nodes are > 3cm in largest dimension, or if extra-nodal cancer extension is present, and if distant metastases are present. The presence of these high risk features in a PTM influences the extent of surgery required for treatment [9]. Small PTM size (< 5mm versus ≥ 5mm) has also been suggested as being important for risk stratification, with some reports identifying PTMs ≥ 5mm as being more likely to have high risk features [8, 10, 11].

The objective of this study was to determine if small PTMs (size < 5mm) are clinically and pathologically different from larger PTMs (size 5mm- ≤ 10mm).

## Methods

The study protocol was approved by our Research Ethics Board. This study was a retrospective analysis of data collected prospectively between 2002 and 2012 for 1459 sequential patients undergoing thyroid surgery at St. Paul’s Hospital, a single tertiary care/academic teaching hospital in Vancouver, Canada. Analysis was limited to cases where pathology identified a PTM of ≤ 1cm *without* the presence of an associated macrocarcinoma (> 1cm). Applying this inclusion and exclusion criteria resulted in 132 cases for analysis. This cohort was then examined based on four clinical and five histopathological characteristics. Clinical variables evaluated were: age (< 45 versus ≥ 45 years), gender (male versus female), surgery performed (total thyroidectomy versus thyroid lobectomy), and surgical indication (benign disease versus known or suspected cancer). Surgical indication was based on pre-operative fine need aspiration (FNAB) and/or imaging findings. Diagnosis of suspicion of malignancy was based upon FNAB diagnosis of nodules, or pre-operative imaging suspicious for local or distant metastases. Histopathological variables evaluated were: cancer multifocality, cancer bilaterality, extra-thyroidal cancer extension, and the presence of local and distant cancer metastases, all based on histopathological reports. Cancers were considered multifocal if ≥ 2 foci were found in one or both thyroid lobes. Size in millimeters (< 5mm vs ≥ 5mm) was the independent variable. The subtype histology of the PTMs were not available for all cases and was not included in the overall analysis.

### Statistical analysis

Statistical differences between groups were determined using the Chi Square and Fisher’s exact tests as appropriate, with a *p*-value < 0.05 considered statistically different and a priori. Analysis was performed in *R*.

## Results

One hundred thirty-two of 1459 patients (9%) met study inclusion criteria and made up the study patient population.

### Clinical characteristics

The clinical characteristics and treatment of the study patient population is summarized in Table 1. Of the 132 patients included, there were 105 (80%) women and 27 (20%) men. The mean age at the time of their procedure was 50.2 years (range 23–74 years) with 48 patients (36%) being less than 45 years old and 84 patients (64%) being aged 45 years or older. The surgical procedure was a total thyroidectomy for 63 patients (48%) and thyroid lobectomy for 69 patients (52%). Indication for surgery was for presumed benign disease in 33 cases (25%) and for suspicion of or confirmed malignant disease in 99 cases (75%). The suspicion of malignancy was generally due to an intermediate Fine needle aspiration biopsy of an associated thyroid nodule > 1cm. Benign pathology included goiter, Hashimoto’s thyroiditis, thyroid cyst, hyperplastic nodules, and Hurthe cell adenomas, all with PTM that was identified incidentally. All cases with confirmed malignancy pre-operatively were papillary thyroid cancer.

**Table 1:** Papillary Microcarcinoma Patient and Operative Characteristics

Patient and operative characteristics	< 5mm *n* = 75	≥ 5mm *n* = 57	Total *N* = 132	Chi square
*Age*				
<45	22	26	48 (36%)	*p* = 0.08
≥45	53	31	84 (64%)	
*Gender*				
Male	11	16	27 (20%)	*p* = 0.09
Female	64	41	105 (80%)	
*Surgery*				
Total Thyroidectomy	37	26	63 (48%)	*p* = 0.80
Thyroid Lobectomy	38	31	69 (52%)	
*Surgical Indication*				
Benign	20	13	33 (25%)	*p* = 0.76
Malignant	55	44	99 (75%)	

Benign pathology included goiter, Hashimoto’s thyroiditis, thyroid cyst, hyperplastic nodules, and Hurthe cell adenomas associated with papillary thyroid microcarcinoma. Malignant pathology (confirmed or suspected) was based on fine needle aspiration biopsy of nodules or pre-operative imaging. All cases with confirmed malignancy pre-operatively were papillary thyroid cancer

At least 1 histopathologic high risk feature was identified in 45 patients (34%). Twenty-seven patients (20%) had 1 high-risk feature, 14 patients (11%) had 2 high risk features and 4 (3%) had 3 high risk features. No patients had 4 or 5 high risk histopathologic features. Multifocality was identified in 29 patients (28%), bilaterality in 8 patients (6% of total, 27% with multifocal disease), extrathyroidal cancer extension in 6 patients (5%), lymph node metastases present at diagnosis in 9 patients (7%) and distant metastases present at diagnosis in 1 patient (0.7%). The study population’s characteristics are summarized in Table 2.

**Table 2:** High Risk Characteristics of Papillary Thyroid Microcarcinoma Patient Population

High Risk Feature	<5 mm *n* = 75(%)	≥ 5mm *n* = 57(%)	Total (% of 132)	Chi square/Fisher’s
Cancer Bilaterality	6 (8)	2 (4)	8 (6)	0.48
Cancer Multifocality	21(28)	17 (30)	38 (29)	0.97
Extrathyroidal Cancer Extension	0 (0)	6 (11)	6 (5)	*p* = 0.005
Lymph Node Metastases	5 (7)	4 (7)	9 (7)	0.78
Distant Metastases	1 (1)	0 (0)	1 (0.7)	0.89
*Total*	24	29	53	

Chi square or Fisher’s Exact test comparison for patient and surgical variables of interest failed to identify a difference between small or large PTM (Table 1). Examination of high risk features revealed that extrathyroidal cancer extension was present significantly more often in large PTM (*p* = 0.005). Other high risk features were not differentially identified based on cancer size (Table 2). The 9 patients who had lymph node metastases present at the time of their cancer diagnosis were further studied. Six of nine patients (67%) had bilateral disease and two of the six also had extrathyroidal extension (both large PTM), while two others had associated bilateral disease (both small PTM), including one patient with distant metastases from a 3mm cancer. Three of the nine patients (33%) had lymph node metastases in the absence of any high risk features (Table 3).

**Table 3:** Patient Population With Lymph Node Metastases

*Pt*	*Age*	*Size mm*	*Histology*	*Bilateral*	*Multifocal*	*ETE*	*LN Mets*	*Dis* *Mets*
**7**	**< 45**	**9**	**PTMC**	**–**	**–**	–	**+**	–
1	< 45	8	PTMCvF	–	+	+	+	–
**8**	≥ **45**	**6**	**PTMC**	–	–	–	**+**	–
3	≥ 45	5	PTMC	–	+	+	+	–
2	≥ 45	3	PTMCvS	+	+	–	+	–
5	< 45	3	PTMC	–	+	–	+	–
6	≥ 45	3	PTMCvF	+	+	–	+	+
4	≥ 45	2.5	PTMCvS	–	+	–	+	–
**9**	≥ **45**	**2**	**PMTCvF**	–	–	–	+	–

*Pt* patient, Bilateral bilaterality, *Multifocal* multifocality, *ETE* extra-thyroidal extension, *LN* Mets lymph node metastasis, *Dis Mets* distant metastasis, *PTMC* papillary thyroid microcarcinoma, *vF* variant follicular, *vS* variant sclerosing

+, present; -, absent

Bold values, LN metastasis in absence of any other high risk features

## Discussion

PTM diagnosis is very common with almost half of all new papillary thyroid cancer (PTC) diagnoses being a PTM. Following thyroid surgery, pathologic identification of PTM ranges from 1–24% of surgical specimens [2, 5, 10], and 6–36% of autopsy specimens [2, 5]. Analysis of the Surveillance, Epidemiology and End Results database revealed that PTM in patients older than 45 years is now the most commonly diagnosed PTC [1]. Our observed rate of PTM diagnosed from surgical specimens in the absence of a thyroid macrocarcinoma was 9%, and was consistent with Canadian autopsy (6%) [12], international autopsy (3–18%) [2] and pathology data (1–24%) depending on the extent of surgical resection [2, 11].

Guidelines regarding the surgical management of PTC have continued to evolve and are currently based on clinical and pathological characteristics of the diagnosed individual and their thyroid cancer, respectively [9}. However, debate still exists regarding whether PTM should be managed in a manner similar to larger PTC [4]. PTM usually has an excellent prognosis and current American Thyroid Association Guidelines recommend that thyroid lobectomy alone is adequate treatment for unifocal intrathyroiodal cancers in the absence of a prior history of head and neck irradiation, familial thyroid carcinoma, or clinically detectable cervical metastases. As there are no features of PTM that can reliably identify the small subset of patients who will go on to develop clinically significant cancer progression, an active surveillance approach that involves close clinical and ultrasound PTM follow up, has currently not been adopted by the vast majority of centers [9]. An observational study, with non-surgical management of PTM, reported 3mm of growth in 6% of patients, with 1.4% of patients developing lateral lymph node metastases at 5 years of follow-up. These authors have suggested non-operative management of PTM in the absence of high risk features [13]. However, other groups have encouraged a surgical approach, which has shifted from thyroid lobectomy to total thyroidectomy, and some groups even recommend nodal dissection of the central neck. Proponents of the latter approach recognize the relationship between high risk features and disease recurrence [14, 15].

High risk features are often present in PTM and one-third of our patients had at least 1 high risk feature identified after pathological examination. While distant metastases were uncommon in our series (< 1%), consistent with other reports [6], multifocality was observed in almost a third of cases (28%) and 27% of these cases had bilateral disease. Reported multifocality rates for PTM have ranged from 15–43% [2, 15], with contralateral malignancy identified in up to 53% of cases [16]. Interestingly, multifocality is believed to reflect multiple independent tumors of different clonal origin, as opposed to intraglandular cancer metastases [17]. While the incidence of PTC multifocality is not related to tumor size [18] as we observed in the current study, it has been reported to be an independent risk factor for cancer recurrence [6, 17]. However, the prognostic significance of contralateral PTM is controversial. Given that only 5–10% of the PTCs recur, some investigators have suggested that recurrence can be treated when detected clinically, while other investigators have argued that reoperative surgery carries additional risks that need to be weighted appropriately [14]. The presence of lymph node metastases at the time of diagnosis also influences cancer recurrence risk and has been reported in 5–51% of cases [2, 15]. The current study observed a 7% rate of nodal disease at the time of PTC diagnosis is at the lower end of this range, but is consistent with the 9% nodal disease risk that has been recently associated with PTM [15]. This low rate of lymph node metastases may be an underestimate as the practice of central neck dissection for these cases is variable. Long-term follow-up of PTM has found that the presence of multifocality and lymph node metastases at diagnoses increases the risk of developing subsequent nodal disease, 11% of multifocal PTM recurred compared with 4% of unifocal PTM tumors [19].

Three of nine patients diagnosed with lymph node metastases at the time of their diagnosis had no other high risk features present. The presence of nodal metastasis in association with PTM is believed to represent different underlying cancer biology when compared with PTM not associated with nodal metastases [5]. Aggressive biological behavior may be related to molecular characteristics not yet well understood. Recent reports have identified molecular BRAF mutations, present in 17–52% of PTM, as potential early genetic lesions in PTC [2, 20]. Presence of BRAF mutations are associated with high risk features, including extrathyroidal cancer extension (ETE) and multifocality, and are also predictive of an increased risk of lateral compartment nodal disease [20]. Lin and colleagues have suggested that pre-operative screening for BRAF mutation using fine needle aspirate biopsy could potentially guide the initial treatment of PTM, with positive BRAF status requiring more aggressive therapy [20]. Other recent studies have found that combining BRAF mutations with a panel of other molecular prognosticators may improve its clinical utility [9].

Some investigators have reported that the tumor size of PTM (< 5 vs ≥ 5mm) may be related to aggressive cancer behavior, with larger PTMs being more likely to have high risk features and nodal metastasis [10, 21]. Other groups have found no relationship between PTM size and the presence of high risk features [22, 23]. Our study found that the size of PTM had little relationship with the presence of high risk features. Only ETE was significantly more commonly present in larger than smaller PTM. While the overall rate of ETE was low (5%), these observations were consistent with prior reports (2–21%) [2, 5]. Kim et al. [24] found that the presence of ETE was directly related to the PTM size but not with the risk of cancer recurrence. Other groups have evaluated the relationship between cancer size and high risk features concluded that PTM size did not influence patient outcomes [10, 21].

The current study has several limitations. It is a retrospective analysis from a single institution and results may not be generalizable to all populations. However, the prospective data collection from a multi-cultural catchment area in a publically funded health care system provides support of the representative nature of the sample. The incidental identification of PTC after surgery for benign disease implies that lymph nodes could be under-sampled, and presence of metastatic disease may be higher than our results suggest. As well, outcome and follow-up data is not available and thus no prognostic information can be inferred from this analysis.

## Conclusions

The diagnosis of PTM after thyroid surgery presents the thyroid cancer management team with a dilemma. Neither PTM size, nor the absence of high risk features, excluded the possibility of synchronous lymph node metastases. While some argue that high risk features should prompt surgical management consistent with PTC >1cm, further study that includes the evaluation of the role of thyroid cancer molecular prognostications is required in order to identify the optimal management algorithm for this common endocrine malignancy.

## Abbreviations

PTM: Papillary thyroid microcarcinoma
PTC: Papillary thyroid cancer
ETE: Extrathyroidal cancer extension

## References

[1] Hughes DT, Haymart MR, Miller BS (2011). The most commonly occurring papillary thyroid cancer in the United States is now a microcarcinoma in a patient older than 45 years. Thyroid.

[2] Pacini F (2012). Thyroid microcarcinoma. Best Pract Res Clin Endocrinol Metab.

[3] World Health Organization (2004). Classificiation of Tumours: Pathology and Genetics of Tumours of the Endocrine Organs.

[4] Plzák J, Astl J, Psychogios G (2013). Current treatment strategies for papillary thyroid microcarcinoma. HNO.

[5] Pazaitout-Panayoitou K, Capezzone M, Pacini F (2007). Clinical features and therapeutic implication of papillary thyroid microcarcinoma. Thyroid.

[6] Roti E, Degli Uberti EC, Bondanelli M (2008). Thyroid papillary microcarcinoma: a descriptive and meta-analysis study. Eur J Endocrinol.

[7] Pellegriti G, Scollo C, Lumera G (2004). Clinical behavior and outcome of papillary thyroid cancers smaller than 1.5 cm in diameter: study of 299 cases. J Clin Endocrinol Metab.

[8] Malandrino P, Pellegriti G, Attard M (2013). Papillary thyroid microcarcinomas: A comparative study of the characteristics and risk factors at presentation in two cancer registries. J Clin Endocrinol Metabl.

[9] Haugen E, Alexander E, Bible K (2015). American Thyroid Association Management Guidelines for Adult Patients with Thyroid Nodules and Differentiated Thyroid Cancer. Thyroid.

[10] Wada N, Duh QY, Sugino K (2003). Lymph node metastasis from 259 papillary thyroid microcarcinomas: frequency, pattern of occurrence and recurrence, and optimal strategy for neck dissection. Ann Surg.

[11] Lombardi C, Bellantone R, De Crea C (2010). Papillary thyroid microcarcinoma: Extrathyroidal extension, lymph node metaseases, and risk factors for recurrence in a high prevalence of goiter area. World J Surg.

[12] Fukanaga FH, Yatani R (1975). Geographic patholgy of occult thyroid carcinomas. Cancer.

[13] Ito Y, Uruno T, Nakano K (2003). An observational trial without surgical treatment in patients with papillary microcarcinoma of the thyroid. Thyroid.

[14] Ricci JA, Alfonso AE (2003). Multifocal micropapillary thyroid cancer: A new indication for total thyroidectomy?. Am Surg.

[15] Karatzas T, Vaseileidadis I, Kapetanakis S (2013). Risk factors contributing to the difference in prognosis for papillary versus micropapillary thyroid carcinoma. Am J Surg.

[16] Pitt SC, Sippel RS, Chen H (2009). Contralateral papillary thyroid cancer: does size matter?. Am J Surg.

[17] Bansal M, Mantha G, Nikforov YE (2010). Molecular and histopathological features of multifocal papillary thyroid cancinomas. Mod Pathol.

[18] Arora N, Turbendian HK, Kato MA (2009). Papillary thyroid carcinoma and microcarcinoma: is there a need to distinguish the two?. Thyroid.

[19] Hay ID, Hutchinson ME, Gonzalez-Losada T (2008). Papillary thyroid microcarcinoma: a study of 900 cases observed in a 60-year period. Surgery.

[20] Lin KL, Wang OC, Zhang XH (2010). The BRAF mutation is predictive of aggressive clinicopathological characteristics in papillary thryoid microcarcinoma. Ann Surg Onocol.

[21] Lo CY, Chan WF, Lang BH (2006). Papillary microcarcinoma: is there any difference between clinically overt and occult tumors?. World J Surg.

[22] Chow SM, Law SC, Chan JK (2003). Papillary microcarcinoma of the thyroid - Prognostic significance of lymph node metastases and multifocality. Cancer.

[23] Pellizzo MR, Boschin IM, Toniato A (2004). Natural history, diagnosis, treatment and outcome of papillary thyroid microcarcinoma (PMTC): a mono-institutional 12-year experience. Nucl Med Commun.

[24] Kim JM, Lee YY (2010). Choi, et al. The clinical importance of minimal extrathyroid extension on tumor recurrence in patients with papillary thyroid carcinoma. Endocrinol Metab.

